# Supplementary material for: Early Renal Remission Is Associated with Increased Likelihood of Subsequent Remission in Lupus Nephritis: Single-Centre Observational Study in Australia
Source: Int J Mol Sci. 2025 Oct 2;26(19):9634. doi: 10.3390/ijms26199634 (PMC12525387; doi:10.3390/ijms26199634)
Supplement: Supplementary file 1 [file ijms-26-09634-s001.zip › ijms-3809048-supplementary.pdf]

International Journal of *Molecular Sciences*

MDPI

Table S1. Association of CRR achievement at 24 months and CRR status at 6 and 12 months in univariable and multivariable logistic analysis

	Unadjusted OR for 24m CRR		Adjusted OR[1] for 24m CRR		Adjusted OR[1] for 24m CRR	
	**OR (95% CI)**	**p-value**	**OR (95% CI)**	**p-value**	**OR (95% CI)**	**p-value**
**6 months CRR status**						
**Non-responder**	1.00		1.00			
**Responder**	9.60 (2.66, 34.67)	0.001	11.23 (2.52, 49.87)	<0.001		
**12 months CRR status**						
**Non-responder**	1.00				1.00	
**Responder**	9.45 (2.62, 34.07)	0.001			11.39 (2.41, 53.80)	0.002
**Age category**						
**≤20**	1.00		1.00		1.00	
**20-40**	1.26 (0.36, 4.43)	0.71	2.08 (0.44, 9.96)	0.36	0.64 (0.12, 3.42)	0.60
**>40**	3.21 (0.66, 15.59)	0.15	20.93 (1.44, 303)	0.026	4.55 (0.32, 64.72)	0.26
**Antimalarial drugs use**						
**No**	1.00		1.00		1.00	
**Yes**	8.00 (0.92, 69.21)	0.059	16.43 (1.06, 255.15)	0.046	15.05 (0.85, 265.75)	0.064

[1]*ORs adjusted for age (category <20,20-40,>40) and antimalarial drugs use*

Table S2. Association of CRR achievement at 12 months and CRR status at 6 months in univariable and multivariable logistic analysis

	Unadjusted OR for 12m CRR		Adjusted[1] OR for 12m CRR	
	**OR (95% CI)**	**p-value**	**OR (95% CI)**	**p-value**
**6 months CRR status**				
**Non-responder**	1.00		1.00	
**Responder**	6.92 (2.06, 23.26)	0.002	9.72 (2.19, 43.03)	0.003
**Age category**				
**≤20**	1.00		1.00	
**20-40**	5.2 (1.32-20.49)	0.018	8.18 (1.51, 44.27)	0.015
**>40**	3.25 (0.66-15.98)	0.15	5.18 (0.66, 40.59)	0.118
**Antimalarial drugs use**				
**No**	1.00		1.00	
**Yes**	1.28 (0.26-6.34)	0.76	0.99 (0.13, 7.39)	0.996

[1]*ORs adjusted for age (category <20,20-40,>40) and antimalarial drugs use*

Table S3. Association of CRR achievement at 24 months with anti-double-stranded DNA (dsDNA) antibody level and C3/C4 levels at 6 and 12 months in univariable logistic analysis

	12m CRR achievement OR (95%CI), p-value	24m CRR achievement OR (95%CI), p-value
**dsDNA level at baseline (+1 IU/mL)**	1.00 (1.00, 1.00), 0.15	1.00 (1.00, 1.00), 0.36
**dsDNA level at 6 months (+1 IU/mL)**	1.01 (1.00, 1.00), 0.47	1.00 (0.99, 1.00), 0.31
**dsDNA level at 12 months (+1 IU/mL)**		1.00 (1.00, 1.00), 0.69
**dsDNA negative at 6 months**	1.00	1.00
**dsDNA positive at 6 months**	0.61 (0.09, 4.01), 0.61	2.87 (0.28, 29.68), 0.38
**dsDNA negative at 12 months**		1.00
**dsDNA positive at 12 months**		1.32 (0.20, 8.64), 0.77
**C3 level at baseline**	0.55 (0.08, 3.86), 0.55	0.33 (0.04, 2.45), 0.28
**C3 level at 6 months**	2.00 (0.18, 22.26), 0.57	2.47 (0.19, 32.43), 0.49
**C3 level at 12 months**		2.28 (0.18, 28.21), 0.52
**-**		
**C4 level at baseline**	0.01 (0.00, 8.24), 0.17	0.01 (0.00, 11.84), 0.20
**C4 level at 6 months**	2.13 (0.03, 140.11), 0.72	0.11 (0.00, 27.31), 0.43
**C4 level at 12 months**		0.66 (0.00, 1568.73), 0.92

Table S4. Subsequent kidney function in 6 months and 12 months complete renal responders

	6months complete responders *RC* (95% CI), p-value*	12months complete responders *RC* (95% CI), p-value*
eGFR difference between:		
6 & 12 months	6.69 (-0.45, 13.84), 0.080	
6 & 24 months	8.29 (0.48, 16.09), 0.038	
12 & 24 months	-0.20 (-4.79, 4.39), 0.93	0.96 (-3.32, 5.25), 0.65
UPCR difference between:		
6 & 12 months	0.09 (0.01, 0.17), 0.026	
6 & 24 months	0.04 (-0.08, 0.15), 0.52	
12 & 24 months	-0.05 (-0.18, 0.08), 0.45	-0.01 (-0.14, 0.13), 0.91
